# Death of Morgan G. Lewis, M. D.

**Published:** 1858-03

**Authors:** 


*Death of Morgan G. Lewis, M. D.*—In the present Journal we give the minutes of the annual meeting of the Erie Co. Medical Society, held in January last. Among those who were present upon that occasion, was Dr. M. G. Lewis, of Black Rock, who was in apparent health at the time, and with every prospect, as far as human eye could discover, of mingling with many successive anniversaries of the society.

But although so short a time has elapsed, Dr. Lewis has paid the debt of nature, and he now sleeps the sleep which knows no awakening this side of the resurrection morn.

Dr. Morgan G. Lewis died at his residence in Black Rock, on Monday, the 8th of February. The announcement will cause an emotion of sorrow in all who knew his worth as a physician and a citizen, and a deeper feeling in those who were intimately connected with him in the social circle. His worth demands at our hands some notice.

Born in Buffalo on the 15th of January, 1813, Dr. Lewis grew up, lived and died in his native place. As he approached the verge of manhood, he studied the profession of medicine with the venerable Moses Bristol. After completing his education at medical institutions in Ohio and Philadelphia, he commenced the practice of medicine at Black Rock, in the spring of 1836, and at the same time assumed the editorship of the Black Rock *Advocate*, a paper then published under the auspices of Gen. Porter, and designed to advance the interests of that place against the village of Buffalo. When the rapid growth of the embryo city made it evident that this contest was a vain one, Gen. Porter removed to Niagara Falls, and for some time after that removal Dr. Lewis edited both the Advocate and the Niagara Falls Journal, still attending, however, to his practice of medicine. Finally abandoning the editorial chair, he devoted himself entirely to his profession, and continued in practice up to the time of his death. He never forgot his interest in public matters, and was always a warm advocate of any plan to advance the interests of the community in which he lived.

It is as a physician only that we, like most others, have known Dr. Lewis. as a practitioner he was a sensible, practical and successful man. In all the associated enterprises of the profession he was in the advance. Always a devoted member of the Erie County Medical Society, he was ready to sacrifice his own personal interests to what he saw was required by the dignity of the society, and did so on more than one occasion with a quiet modesty which endeared him to his colleagues. He never placed money before his duty to his profession, and twice within our knowledge has refused offices which he thought inconsistent with the dignity of a physician.

When any individual cherishes this high sense of honor in his intercourse among his fellows, we may look to him as a man of generous and charitable impulses. No other evidence is needed of the possession of these qualities by Dr. Lewis, than the universal grief among the poor of Black Rock which followed his death. On Monday the door of his house was beset by many poor women who came with anxious inquiries and went away with tears for the loss of a kind and helpful friend.

Dr. Lewis was married about three years since. A little child, the only fruit of this union, died about two weeks since, and Mrs. L. is left doubly bereaved. As the hour of death approached, Dr. Lewis recognized its coming, and with the courage of a true and consistent christian, gave verbal directions for the arrangement of his funeral, and the disposition of his affairs Even the possible desire of his physicians for an autopsy was anticipated, and a wish expressed that should be such a measure deemed useful it should be permitted.

For the purpose of paying a proper tribute of respect to the memory of the deceased, on the evening of Wednesday, Feb. 10th, a meeting of the Medical Society of the county of Erie, was held at the rooms of the Buffalo Medical Association, Prof. Austin Flint, president, in the chair, and James M. Newman, secretary. The meeting was largely attended.

The president announced the object of the meeting.

Prof. James P. White announced the death of Dr. Morgan G. Lewis. He said that he rose to express his own regret, and to ask that a committee of three be appointed to draft resolutions expressive of the feelings of the society. Dr. White paid an earnest tribute to the singular purity of the moral and professional character of Dr. Lewis, and to his excellence as a physician.

Dr. Phineas H. Strong seconded the motion in a few remarks bearing on the consistent and amiable character of Dr. Lewis.

Prof. Thomas F. Rochester spoke with feeling of the circumstances attending the final illness of Dr. Lewis, and the enduring love of his profession, which continued to his latest hour.

The president then appoiuted Drs. Rochester, White and Wilcox, as the committee to report resolutions.

Dr. Rochester, from the committee, reported as follows:

*Resolved*, Inasmuch as it has pleased Almighty God to remove from us our friend and associate, Dr. Morgan G. Lewis, as fellow citizens and members of the Erie County Medical Society, we mourn the loss of a companion endeared to us by professional and private worth of the most enviable character. A native of Buffalo, and a practitioner in its immediate vicinity for more than twenty years, he was universally respected and esteemed by all who knew him. Quiet and retiring in his manners, entire kindness and reliability were not less characteristic than modest demeanor;—a beloved physician, cherished friend, excellent citizen, and humble and devoted Christian, his death is no ordinary bereavement.

*Resolved*, That we tender our respectful sympathy to his widow, to his aged mother, and to his other relations.

*Resolved*, That we will attend his funeral in a body, and wear the usual badge of mourning.

*Resolved*, That the foregoing resolutions be sent to his family, and published in the Buffalo Medical Journal and daily papers of this city.

In accordance with a wish expressed by the family of the deceased, Drs. Flint, Rochester, Hamilton, Strong, Wilcox and L. P. Dayton, were appointed pall-bearers

The society then adjourned.

A meeting of the Buffalo Medical Association had been appointed for this evening, but it was adjourned out of respect for the deceased, as he had been always a firm and warm member and friend of the same, although but seldom able to attend its meetings, owing to the distance of his residence.

